# Dinner Date: Opposite-Sex Pairs of Fruit Bats (*Rousettus aegyptiacus*) Forage More than Same-Sex Pairs

**DOI:** 10.3390/biology14121742

**Published:** 2025-12-05

**Authors:** Alexander Pergams, Mara Haus, Cristina Estrada, Joel Brown, Angeles Salles

**Affiliations:** 1 Department of Biological Sciences, University of Illinois Chicago, Chicago, IL 60607, USA; alex@pergams.com (A.P.); mhaus@uic.edu (M.H.); cestr4@uic.edu (C.E.); squirrel@darwiniandynamics.org (J.B.); 2 Department of Biological Sciences, Harper College, Palatine, IL 60067, USA; 3 Integrated Mathematical Oncology, Moffitt Cancer Center, Tampa, FL 33612, USA; 4 Department of Psychology, University of Illinois Chicago, Chicago, IL 60607, USA

**Keywords:** bats, Chiroptera, giving up densities, social foraging

## Abstract

Social animals have unique foraging behaviors that consider not only the needs of the individual, but also the needs of the group. When social animals forage together, there is a greater sense of perceived safety, leading to less vigilance and more foraging. Within these groups, there are consistent differences in foraging across sexes due to inherent differences in behavior and nutritional demands. We hypothesize that social foraging efficiency may be affected by social context, especially by the sex of the individual bats. We used giving-up densities and video data to observe social and asocial foraging in the Egyptian fruit bat (*Rousettus aegyptiacus*) to gain insight into the effect of social context and pairing on foraging. We found that females harvested less food despite spending the same amount of time foraging as males. We found an effect of social context on foraging measures: male–female pairs harvested more than same-sex pairs. Foraging behavior is influenced not only by the sex but also by the identity of the foraging companion. Our study suggests that animals that live in larger groups may require the presence of more individuals while foraging to gain significant perceived safety benefits. It also ascertains the importance of sex in the social and solitary foraging of *Rousettus aegyptiacus* bats.

## Simple Summary

Consistent with previous reports on wild bats, we found that captive Egyptian fruit bats efficiently choose higher-quality food patches to forage from. In our study, mixed-sex pairings of bats had the greatest effect when foraging in a social context. We found that females harvested less food despite spending the same amount of time foraging as males. Our study emphasizes the importance of sex in social and solitary foraging.

## 1. Introduction

Foraging behavior is a complex process. To forage efficiently, animals weigh the validity and importance of information, enabling them to make rapid and continuous decisions [1,2]. In making these decisions, social animals, such as bats, must consider not only individual needs, such as energetic costs, predation risk, and missed-opportunity costs [1], but also the continued survival of the group [3,4]. This weighing of information becomes an even more complex task when considering group dynamics, such as dominance in the social hierarchy [5], and potential mating opportunities [6]. Behavioral decisions are also affected by the individual personality of the forager [7]. Our work explores the effects of sex and pairings on foraging strategies in the social Egyptian fruit bat (*Rousettus aegyptiacus*).

Different species of animals, including bats, will forage from a depletable food patch until the fitness rewards no longer exceed the costs [1,8]. These costs consist of energetic expenditure, predation risk, and missed-opportunity cost [1]. The amount of food left behind after a forager quits a patch is the giving-up density (GUD). Because all animals must forage, GUDs provide an objective method for measuring how an animal perceives its environment. When experimental food patches offer diminishing returns and differ in initial abundances, many species, including Egyptian fruit bats, reveal their ability to assess and respond by consuming a greater proportion of food from rich patches (patches with greater food abundance) than from poor ones, thus equalizing GUDs [9,10,11]. Successful experimental food patches will, by necessity, be species- and context-dependent. Here, we develop an artificial experimental patch for captive Egyptian fruit bats inspired by Sanchez and colleagues [10].

Egyptian fruit bats live in colonies ranging from several to thousands of individuals [12]. Egyptian fruit bats prefer proximity to conspecifics over isolation. They spend more time with members of their own colony than with unfamiliar conspecifics [13]. Social behaviors and abilities, such as individual recognition [13], auditory and olfactory communication [13,14], and even voluntary isolation during illness [15], are thus necessary adaptations for social living in this species. It follows that, like many social bat species [4,16,17], Egyptian fruit bats should also employ social foraging tactics. In many animals, larger groups of conspecifics can detect novel food patches faster by multiplying search efforts and communicating about opportunities [18]. Once said patches are detected, it has been shown for a variety of species that individuals in groups begin foraging more readily and harvest a higher amount of food than individuals foraging solo or in smaller groups due to perceived safety benefits [18,19]. The number of individuals required in a group to increase per capita food consumption varies depending on the species [20].

The sex composition of groups may determine foraging behavior and efficiency. Male and female animals often differ in their foraging behavior, including foraging timing [21,22], habitat use [23,24], and diet [25,26,27,28]. In Egyptian fruit bats, males emerge from the colony caves to forage earlier than females, but only during the pregnancy season [29]. In comparison to males, females also use more native forest patches (rather than human-developed areas) and differ in the proportion of time spent feeding on different food types [30]. The authors show that a similar proportion of males and females used different types of food, but they differed in the proportion of time they spent feeding on different types of food. Females spent more nights feeding on date palm flowers, but spent less time on figs than the males. The most striking difference between sexes in the foraging of Egyptian fruit bats is their scrounging behavior. Scrounging refers to when females approach colony mates (usually males) and take food from their mouths. Scrounging bats have consistent preferences for who they scrounge from [31]. Males can choose to either respond aggressively to these attempts or voluntarily relinquish the food item [6]. Allowing scrounging from females increases mating opportunities for males, as females are more likely to deliver pups for males who allow scrounging [6]. Thus, scrounged food may act as a nuptial gift.

We aim to gain unique insights into the foraging strategies and social perceptions of sex in Egyptian fruit bats by observing the solitary and social foraging behavior of bats paired with the same and different biological sexes. Using experimental food patches in a captive setting, we measured GUDs and foraging strategies of the bats through video observations. We hypothesize that optimal foraging in Egyptian fruit bats changes in response to social context and the sex of individuals. We also hypothesize that competition between males increases GUDs because males would invest time dealing with competitors. We will measure the effect of foraging time, number of times a patch is visited, and sex pairing on GUDs. We predict that males will, on average, forage more than females due to competition with other males, the perceived metabolic cost of potential mating with females, increased metabolic cost, decreased vigilance due to their larger size, and investing harvested food to scrounging by females. Vigilance, in this case, is defined as reduced time spent consuming food and thus attending to the environment. We also predict that bats will change their strategies based on whether they are foraging with a member of the same or opposite sex due to scrounging and competition for mates.

## 2. Methods

### 2.1. Study Animals

Twelve bats were used in these experiments, and each bat was paired with all the other bats. The bats used were six adult males, ranging in mass from 118 to 163 g, and six adult females, ranging in mass from 115.8 to 137.8 g, and they were selected randomly from a captive colony of Egyptian fruit bats consisting of about 25 individuals housed in a large flight room measuring 4.27 × 3.05 × 2.43 m in a lab building of the University of Illinois Chicago, in Chicago, Illinois (protocol #22-056). Each bat was weighed before and after two consecutive foraging trials (two 24 h periods of foraging). All bats were kept on a 12 h/12 h day/night cycle, at temperatures ranging from 20 to 24 °C, and humidity ranging from 60 to 75%. Because of the time of year in which the experiments were conducted, male bats were in the regressed reproductive phase, and females were post-lactating.

### 2.2. Foraging Preference for Rich Versus Poor Patch Qualities

We first created a novel food patch and tested bat foraging preference and patch validity by assessing if bats fed preferentially from patches with greater amounts of food. While artificial food patches generally cannot mimic foraging behavior in nature, similar food patches have been used to successfully assess foraging behavior in fruit bats by achieving diminishing returns [10,11,32]. First, the bats were tested individually on the food patches to establish a preference for the rich patch. Here, each individual was placed in one of two mesh fabric enclosures, each measuring 95 × 95 × 60 cm, with each enclosure’s zippered opening facing the other’s (Supplementary Figure S1).

In each enclosure, we placed two food patches (Supplementary Figure S2) made from translucent plastic containers (measuring 15 × 15 × 9 cm) and inspired by food patches designed and tested by Sánchez and collaborators [10]. The food in these containers consisted of a smoothie made from: 6 bananas, 4 apples, 4 cups of pears, 0.5 cup of mango, 100 g marmoset diet, and 1/2 cantaloupe, blended until liquid. For the substrate of a patch, we placed 11 large plastic beads, each measuring approximately 3 × 3 × 3 cm, tied together with a shoestring. The beads were intended to slow foraging and create diminishing returns to time spent feeding from the patch. The bats had to expend time and effort moving beads to access the food underneath. We then put three grapes of comparable size on top of each patch to entice bats into foraging. In each enclosure, one patch contained 100 g of smoothie (“poor” patch) and the other 200 g of smoothie (“rich” patch). Each patch was weighed in its entirety prior to placement in the enclosure. The weight of the substrate (beads and shoestring) and plastic containers was measured separately. We placed a WYZE Cam v2 camera in each enclosure to provide videos for measuring the number and duration of visits to each patch.

In accordance with the marginal value theorem [33], foragers should expend more effort at the rich patch than at the poor patch. Thus, when offered pairs of patches, one with 100 g and the other with 200 g, the bats will reveal patch choice and diminishing returns by harvesting a higher fraction of food from the rich than the poor patch [9].

While bats were physically separated from each other during parts of this experiment, they could see each other and communicate across the mesh (Supplementary Figure S3). Bats were left to forage for about 24 h on a 12:12 light/dark cycle, in which bats consumed food usually within the first 6 h, as this matched their crepuscular light settings in which feeding occurs. The 24-h initial period was selected to allow the bats to acclimate to the chambers. After this period, all patches were again weighed, cleaned, and the process was repeated for an additional 12 h period. GUDs were calculated as the difference in total mass of the patch after the 24 h period.

### 2.3. Social and Solitary Foraging Across Different Sex Pairings

We next paired bats with conspecifics of the same or opposite biological sex and had them forage in isolation and together to examine the effects of social context on foraging (Supplementary Video S1). Bats were tested in pairs and placed in adjacent fabric enclosures in the experimental room, separated by a closed zipper opening. The artificial food patches from the first experiment were recreated, but this time only one patch was placed in each enclosure, and patches always contained 200 g of smoothie. As before, bats were left to forage for 24 h (each in their own separate but adjacent enclosure), after which food patches were weighed and replenished. As before, GUDs were measured as the difference in the total mass of the patch, minus the mass of the substrate and container. Foraging activity was video recorded.

**Figure 1:**
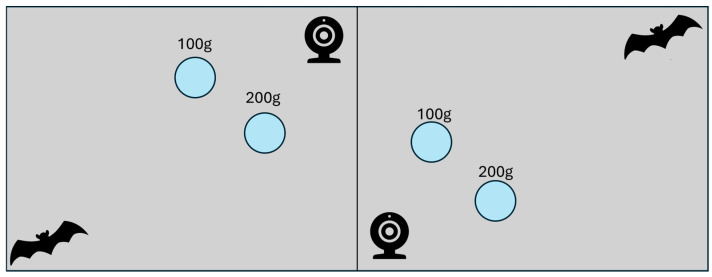
Experiment 2: Testing for effects of social context and sex on foraging times and GUDs. For this experiment, each half of the enclosure had one bat, one WAYZ camera, and one food patch with 200 g of smoothie (blue circle). Bats foraged separately with the divider (middle line) closed for one 24 h trial, after which point the food patches were removed and replaced with fresh ones, and the divider was opened, allowing bats to forage together for a second consecutive 24 h trial.

After the first 24 h period, new food patches were placed in the enclosures, and the zippers were opened on both enclosures, connecting the enclosures and allowing bats to interact directly and access both food patches (Figure 1). This process was repeated until all combinations of the bats had gone through foraging trials together, with no bat spending more than 48 consecutive hours away from their home colony. In this way, we tested a total of three experimental blocks (sets) consisting of four bats each, with all combinations of bats within each block

We used the motion-filtering program DVR-Scan [34] to select periods of motion in the region of interest (the food patches). We then used the behavioral analysis program BORIS, which allows for manual annotation of behavioral video data [35] to record the identity of the bat (one bat of each pair had its ear marked with non-toxic, water-soluble, chalk paint) and the duration of time for each visit to a food patch. We thus gained additional foraging metrics, by individual and by patch, for each trial to supplement GUDs: time spent foraging, number of food patch visits, and mean duration of patch visits. This allowed us to parse out the foraging behavior of individuals during social foraging trials.

### 2.4. Statistical Analysis

All analyses were carried out in R (version 4.1.3) [36]. Normality for all data was determined using the Shapiro–Wilk test. Even though bats could be distinguished by their ear markings, it was impossible to determine the amount each bat consumed from one bowl for the GUDs in a social context. Thus, solitary trials alone were used for analyses that involved comparing the GUDs of individuals. While Shapiro–Wilk tests suggested that only GUD data was normally distributed, normality of model residuals indicated that the linear regressions were appropriate. Effects were considered significant at *p* < 0.05.

For Experiment 1, a paired *t*-test with GUDs as the dependent variable was used to assess bat foraging preference between poor and rich food patches in the bat foraging preference experiment.

For Experiment 2, we used ANOVAs and ANCOVAs on linear models fitted using the lm function to determine correlated metrics. Analyses of covariance (ANCOVA) were used to determine the effect of the number of patch visits and time spent foraging on GUDs with sex as a grouping variable. An ANCOVA was also used to test for the effect of sex and body mass on GUDs. A nested analysis of variance (ANOVA) was used to determine the effect of individuals nested in sex on GUDs. Then, ANOVAs were used to assess whether patch use across the two patches was similarly equal across solitary and paired trials. Finally, ANOVAs were used to determine if bats preferred their initial patch or their neighbor’s patch from the solitary trial during the associated social foraging trial.

We then used generalized linear mixed models (GLMM) to assess the effect of social foraging (presence or absence of a conspecific), pairing (male–male, female–female, or male–female), and sets of four bats on video-recorded foraging metrics (overall time spent foraging, number of visits to a food patch, and mean time of patch visits), with bat ID included as a random effect. We fitted the models to appropriate error structures based on dispersion and distribution of residuals. Of the response variables, overall time spent foraging was fitted to a negative binomial error structure with quadratic parameterization, patch visit number was fitted to a negative binomial error structure with linear parameterization, and mean visit time was fitted to a generalized Poisson error structure. A generalized linear model (GLM) was then implemented to assess the effect of social foraging, pairing, and bat set on GUDs (square root transformation, Gaussian error structure). We used the glmmTMB function [37] to fit these models. We then used the drop1 function [38] to perform likelihood ratio tests (LRTs) to determine the significance of each fixed effect in each model. When response variables were significantly influenced by bat set or pairing, we calculated pairwise comparisons using the emmeans function from the emmeans package (version 1.11.1) [39].

## 3. Results

### 3.1. Egyptian Fruit Bats Prefer to Forage from Rich Food Patches

**Figure 2:**
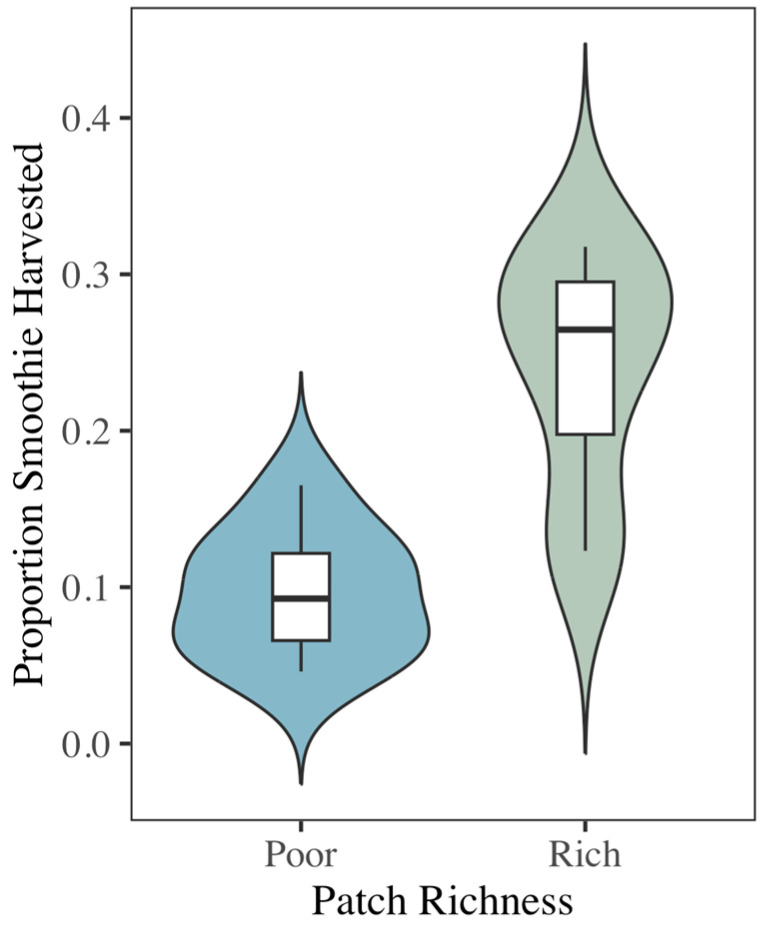
Violin plot of the proportion of smoothie harvested from rich (200 g of smoothie) and poor (100 g of smoothie) food patches. Bats biased their foraging activity towards the higher-quality food patches (paired *t*-test, t_7_ = 4.97, *p* < 0.01). Middle lines in each box represent the median proportion harvested across patches. Upper whisker = Q3 + (1.5 × IQR) and lower whisker = Q1 − (1.5 × IQR). IQR= interquartile range.

Bats preferentially foraged from food patches containing 200 g of smoothie compared to the patches with 100 g. The bats harvested a higher proportion of food from the higher-quality patch (t_7_ = 4.97, *p* < 0.01) (Figure 2). As predicted, both showed that bats were able to bias their foraging towards food patches with the highest benefits and the least cost, demonstrating the effectiveness of our food patches.

**Figure 3:**
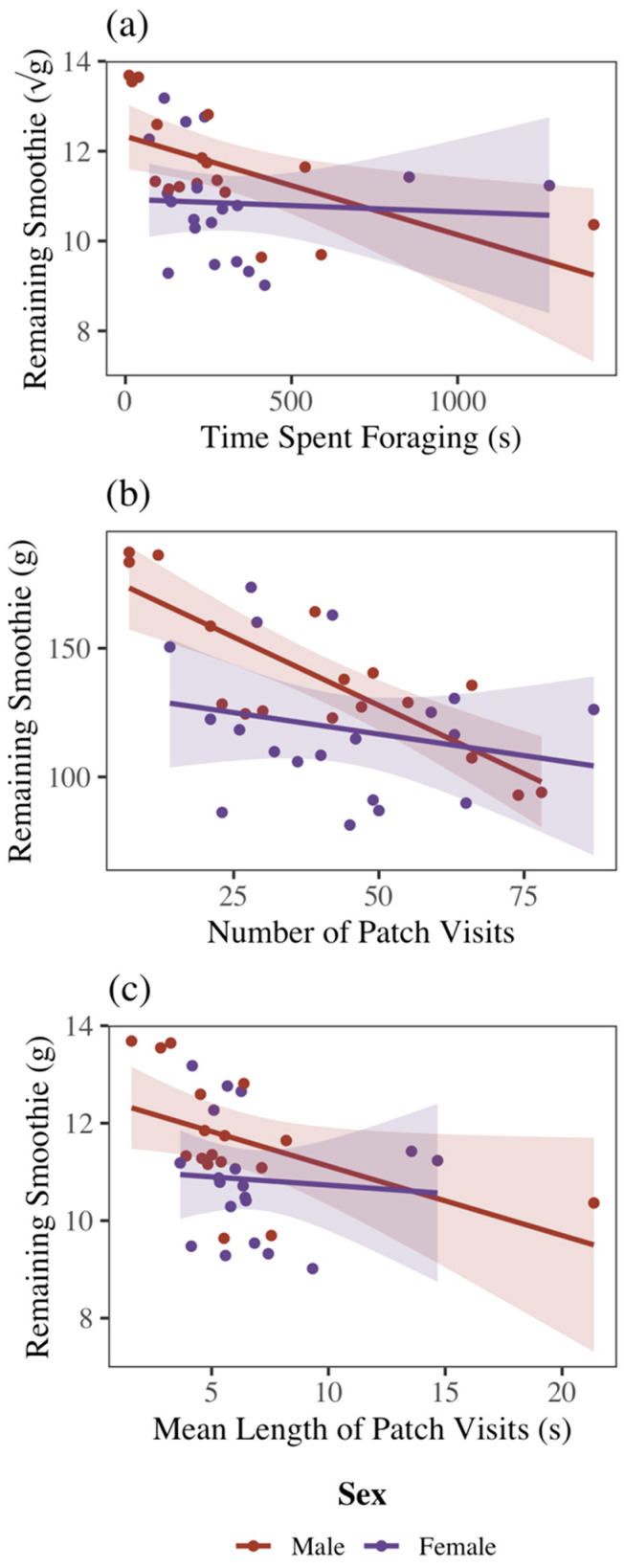
Effect of (**a**) time spent foraging, (**b**) number of visits to food patches, and (**c**) mean length of patch visit on giving-up densities in male and female Egyptian fruit bats. As expected, GUDs (the remaining smoothie) declined with the total time devoted to harvesting a patch. Shaded areas represent standard deviation.

For both sexes pooled together, bats that foraged longer also harvested more food (Table 1; Figure 3a) (F_1,32_ = 4.16, *p* < 0.05) when foraging in isolation. Bats that visited available food patches more frequently also harvested more food (Table 1; Figure 3b) (F_1,32_ = 15.06, *p* < 0.001). The mean length of time a bat visited a patch had no significant effect on the amount of food harvested (Table 1; Figure 3c) (F_1,32_ = 3.47, *p* = 0.07). Hence, the number of visits provides the best metric of reward for foraging effort.

### 3.2. Sex and Foraging Metrics

**Figure 4:**
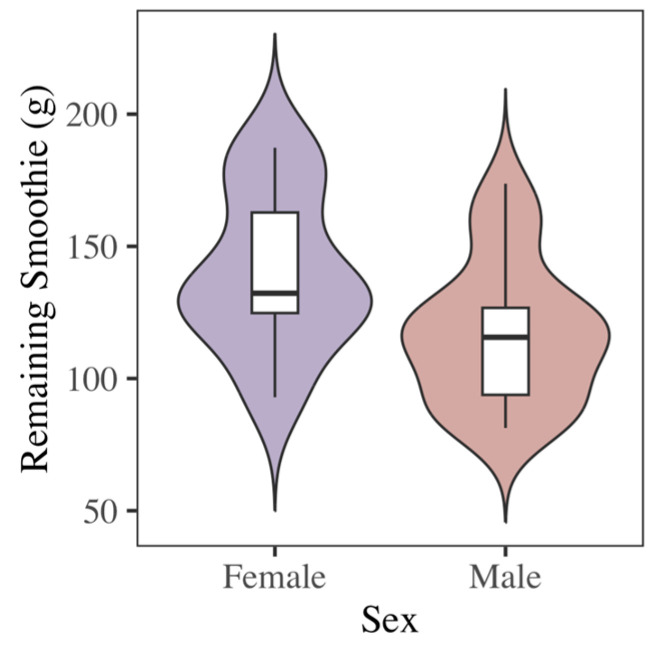
Violin plot showing differences in giving-up densities between male (M) and female (F) Egyptian fruit bats in a solitary context. Upper whisker = Q3 + (1.5 × IQR) and lower whisker = Q1 − (1.5 × IQR). Males harvested significantly more food from patches than females (F_1_ = 8.23, *p* < 0.01).

In solitary foraging trials, male bats harvested more food than females (Figure 4) (F_1,28_ = 8.23, *p* < 0.01). There were no significant interaction effects between sex and foraging time (Table 1) (F_1,32_ = 2.18, *p* = 0.15), sex and number of patch visits (F_1,32_ = 3.49, *p* = 0.07), or sex and mean patch visit time (F_1,32_ = 0.81, *p* = 0.37) on GUDs. In solitary and social trials combined, there was no difference between sexes in total time spent foraging (F_1,68_ = 0.13, *p* = 0.72), number of visits to food patches (F_1,68_ < 0.01, *p* = 0.98), or mean patch visitation time (F_1,68_ = 0.02, *p* = 0.89). However, GUDs declined more quickly in response to all three foraging metrics for females than for males (Figure 4). While there was no individual effect on GUDs (F_10,28_ = 1.86, *p* = 0.09) in solitary foraging trials combined, there was an individual effect on time spent foraging (F_10,60_ = 3.66, *p* < 0.001), number of patch visits (F_10,60_ = 4.09, *p* < 0.001), and mean visit time (F_10,60_ = 3.31, *p* < 0.01).

### 3.3. Sex and Mass

**Figure 5:**
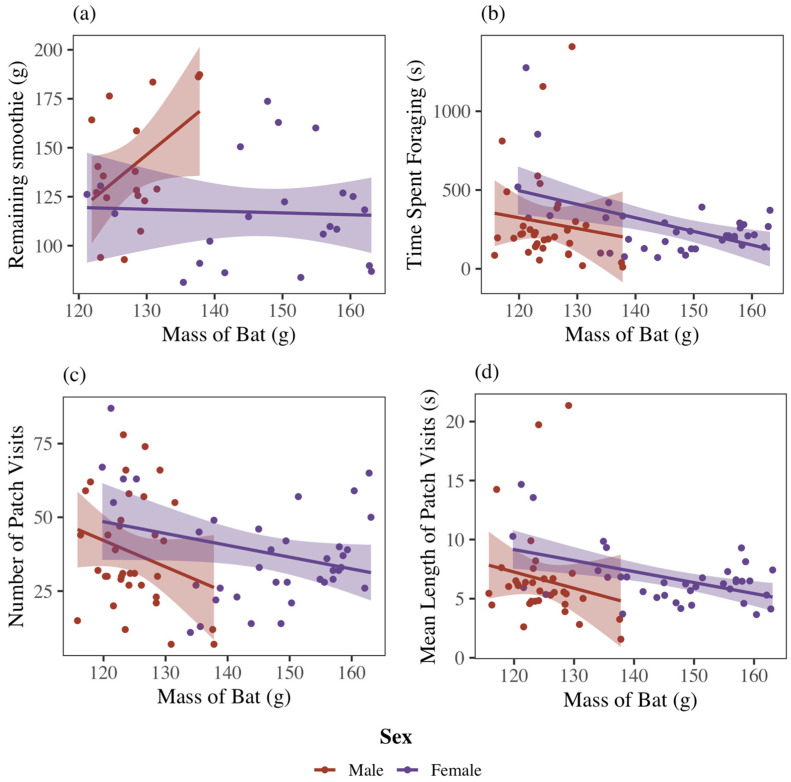
Effect of bat mass on (**a**) giving-up densities, (**b**) time spent foraging, (**c**) number of patch visits, and (**d**) mean length of patch visit in male and female Egyptian fruit bats. All measures except the remaining smoothie for males are negatively correlated with bat mass.

Females (mean mass, 121.2 g; range 115.8–124.3 g) were significantly smaller than males (mean mass, 146.5 g; range 135.6–159.3 g) (t_10_ = −5.50, *p* < 0.001). There was no interaction effect between sex and mass on time spent foraging (Figure 5b) (F_1,68_ = 0.04, *p* = 0.84), number of patch visits (Figure 5c) (F_1,68_ = 0.61, *p* = 0.44), or mean visit time (Figure 5d) (F_1,68_ = 0.15, *p* = 0.70). While not statistically significant (F_1,36_ = 3.95, *p* = 0.05), there was a closer positive association between mass and GUDs in females than in males. In a solitary context, larger females harvested less food than smaller females (Figure 5a). After factoring out the effects of sex, larger individuals spent less time foraging (Figure 5b) (F_1,68_ = 7.41, *p* < 0.01), visited patches fewer times (Figure 5c) (F_1,68_ = 4.69, *p* = 0.03), and had shorter average visits to patches (Figure 5d) (F_1,68_ = 6.38, *p* = 0.01). In a solitary context, mass did not have a significant effect on GUDs (F_1,36_ = 0.2, *p* = 0.66), indicating that larger individuals had faster harvest rates from patches either because their size increased the overall speed, or we can hypothesize that larger individuals may be less vigilant while harvesting food patches.

### 3.4. Solitary vs. Social Foraging

**Figure 6:**
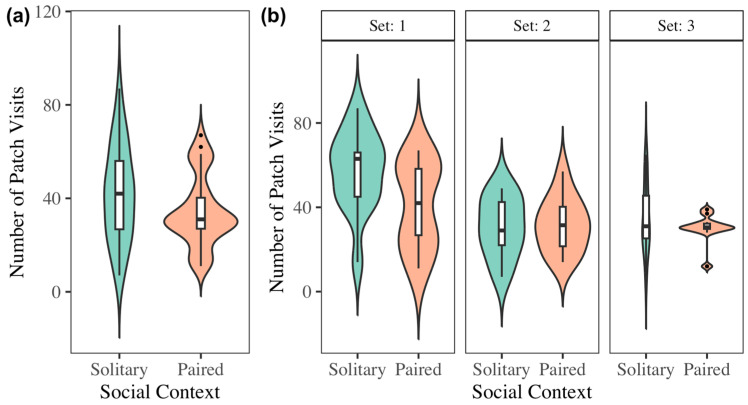
Combined across sets (**a**), bats made more visits to a food patch when foraging in a solitary context than in a social context (F_1,62_ = 4.83, *p* < 0.05). However, when separated by set (**b**), the data showed that this difference was only significant for Set 1.

**Figure 7:**
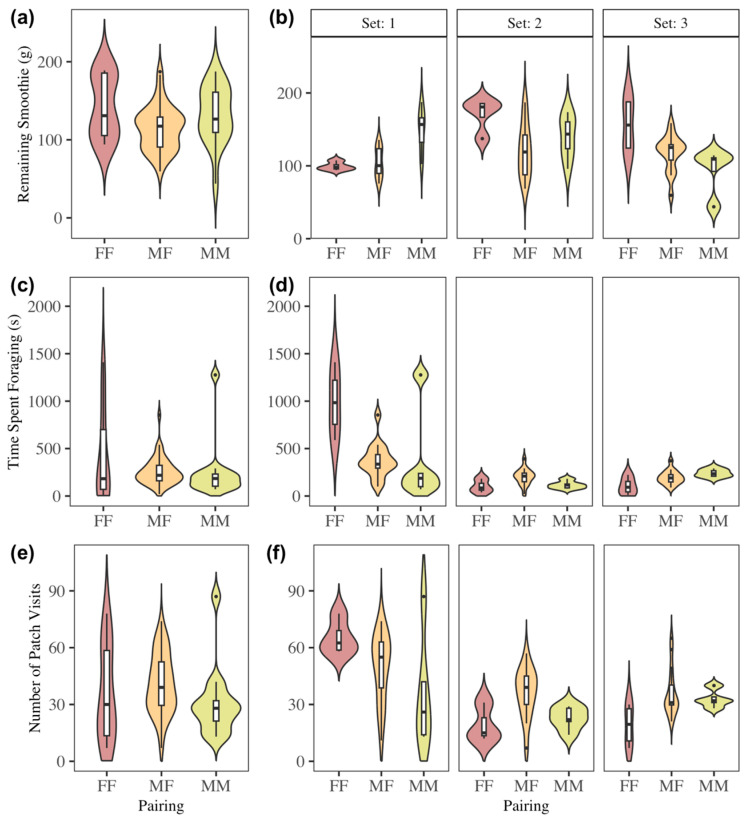
The effect of pairing (male–male, male–female, or female–female) on giving-up densities (**a**,**b**), time spent foraging (**c**,**d**), and number of patch visits (**e**,**f**), both combined across sets of bats (**a**,**c**,**e**) and separated by set (**b**,**d**,**f**). Upper whisker = Q3 + (1.5 × IQR) and lower whisker = Q1 − (1.5 × IQR).

Contrary to our predictions, foraging together with a conspecific had no effect on GUDs, overall time spent foraging, or mean patch visit time (Table 2) compared to foraging separately. Patch visitation frequency decreased in the social context (Table 2) (Figure 6a) for the first experimental set. Set 1 was significantly different from sets 2 and 3 across all response variables except GUDs (Table 2) (Figure 7).

### 3.5. Effect of Pairing on Foraging

The sex of bat pairings had a significant effect on GUDs (Figure 7a; Table 2), patch visit number (Figure 7e), and mean visit time, but not time spent foraging (Figure 7c; Table 2). Opposite-sex pairings had significantly longer and more frequent patch visits compared to male–male pairings (Table 2; Figure 7e). F-F pairings were not significantly different. Meanwhile, opposite sex pairings exhibited significantly lower GUDs when compared with female–female pairings. The presence of females seemed to increase male patch visitation number and length, while the presence of males seemed to increase female harvest rates. Though there was variability across sets of bats when analyzing each separately, the effect was reproducible across sets 2 and 3 (Figure 7b,d,f).

### 3.6. Patch Equity

**Figure 8:**
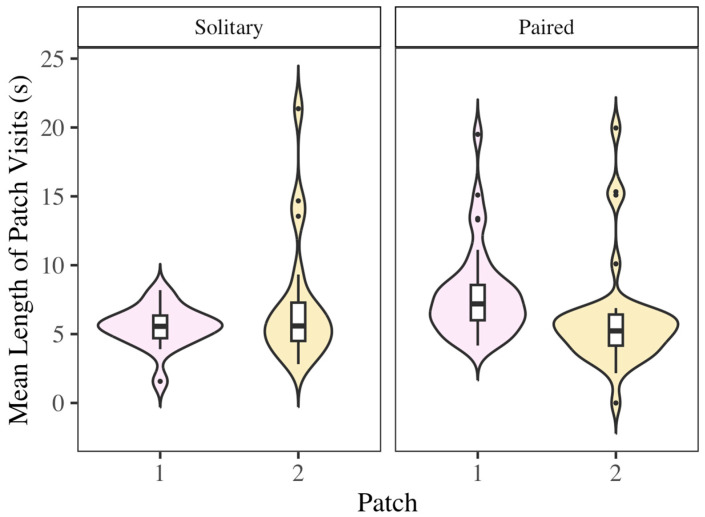
Mean patch visit time was more unequal across patches in a social context than in a solitary context (F_1,104_ = 5.89; *p* < 0.05). Bats demonstrated a preference for one patch or the other when multiple patches were available to them. Upper whisker = Q3 + (1.5 × IQR) and lower whisker = Q1 − (1.5 × IQR).

In a social context, bats were more unequal in their mean patch visit times between patches than in a solitary context (Figure 8) (F_1,104_ = 5.89; *p* < 0.05). This was not the case for time spent foraging (F_1,104_ = 2.02; *p* = 0.16) or number of patch visits (F_1,104_ < 0.01, *p* = 0.98). This suggests that bats changed how they selected patches when they had multiple patches available to them.

### 3.7. Preference of Starting Patch

**Figure 9:**
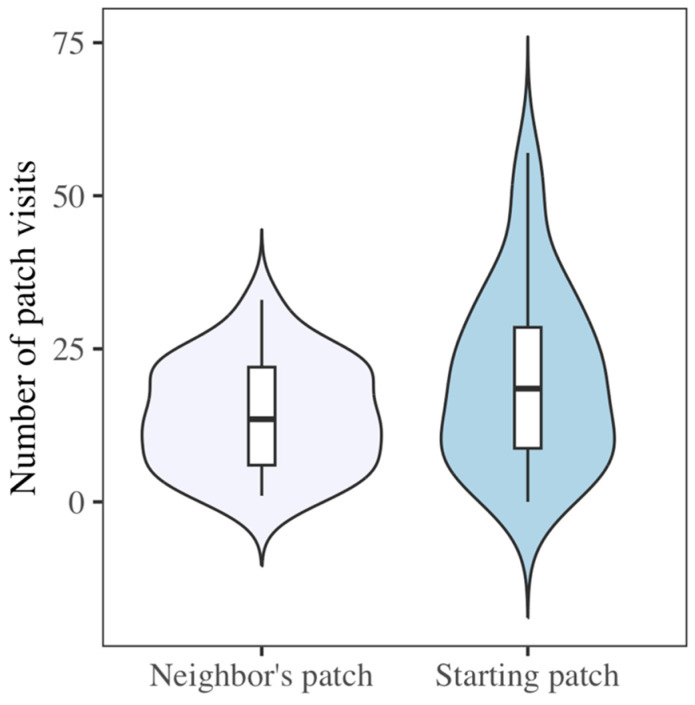
In a social context, bats visited the patch of their solitary trial more often than the newly accessible patch of their neighbor (F_1,70_ = 4.59, *p* < 0.05). Upper whisker = Q3 + (1.5 × IQR) and lower whisker = Q1 − (1.5 × IQR).

In a social context, bats visited their original patch (the patch on their side of the enclosure) more frequently than they visited the newly accessible patch of their neighbor (Figure 9) (F_1,70_ = 4.59, *p* < 0.05), perhaps indicating a sense of familiar versus unfamiliar space. Only the frequency of patch visitation was affected, however, as they did not spend more time at their own patch (F_1,70_ = 2.5, *p* = 0.12) or have a greater mean patch visit time (F_1,70_ = 1.85, *p* = 0.18).

## 4. Discussion

Egyptian fruit bats have complex interactions and display a wide array of social behaviors; our study demonstrated how social context affects their foraging behavior. Fruit bats can bias their foraging to obtain the most benefits for the least cost. The foraging strategies of male and female bats differ, with females foraging the same amount of time as males but harvesting less. Body mass was a strong indicator of foraging behavior. Contrary to predictions, smaller individuals spent more time harvesting food. Bats changed their foraging strategy based on whether they were alone or with a conspecific, but this based on whether they were foraging with a member of the same or opposite sex. Foraging varied based on the set of individuals involved and the sex of the pairing.

### 4.1. Foraging Behavior and Strategies

Egyptian fruit bats were able to distinguish between rich and poor food patches and bias their effort towards the high-quality patches. Despite differences in patch design, our results are consistent with those of Sánchez [10,11], who first developed a novel depletable food patch for Egyptian fruit bats. This suggests not only success in our patch design but also confirms that fruit bats are able to use sensory cues to forage optimally [2,40]. The correlation between foraging time and GUDs was as expected, given previous research [41]. Here, however, patch visitation frequency was a better predictor of food harvested than total foraging time. This suggests that much of the time spent on food patches is not spent on foraging. This could be the result of a high level of task difficulty. In this case, a high proportion of time in the patch was likely spent on being distractedly alert rather than single-mindedly concentrating on foraging [42,43,44].

### 4.2. Sex and Size Differences in Foraging

Female Egyptian fruit bats were more vigilant than males (as indicated by a lower amount of collected food), and smaller bats devoted more total time to patches than larger ones. Females harvested less food than males, but had no significant difference in the amount of time foraging. The lack of difference between sexes in time spent foraging is consistent with that of Wilkinson and Barclay [24] in big brown bats (*Eptesicus fuscus*) and Sealey and collaborators [45] in Jamaican fruit bats (*Artibeus jamaicensis*). Sexual size dimorphism alone [46] does not account for the fact that females harvested less food while spending a similar amount of time foraging, as there was a sex difference even after accounting for size. It instead suggests that females may have been apprehensive, distracted, and vigilant while foraging. Females may thus profit more from the protection of large groups. The increased nutritional demands of pregnancy in the first two females may help explain the greatly increased foraging time by these females in set one compared to females in the other two sets. Egyptian fruit bats have empirically been shown to increase food consumption during pregnancy [47].

Larger bats spent significantly less time foraging than smaller bats. While larger animals require more food for body maintenance [26,48], smaller animals require more nutrients and metabolize energy at a faster rate [49,50]. Smaller male bats may thus have a larger marginal value of energy and may consume more food per unit mass. Altruistic behavior has been recorded in other species of social bats [3], and it is possible that larger individuals forego foraging opportunities to allow feeding by smaller individuals. Alternatively, bats may be acting opportunistically by exploiting feeding instances as they become available. Another possible explanation is that larger individuals were more efficient in extracting the food from under the plastic beads. This hypothesis is confirmed by the fact that the larger individuals spent less time foraging, but collected a comparable amount of food, showing a higher rate of food collection.

### 4.3. Foraging and Social Context

Contrary to expectations, foraging with a conspecific did not have a significant effect on GUDs (amount of food harvested when the forager decided to quit the patch) compared to foraging alone. In the wild, colonies of fruit bats can range from one hundred to several thousand individuals [51,52] but often forage alone. Our experiment did not allow us to test the perceived risk of predation, and other factors may be involved in the responses observed, yet we speculate that having one additional individual may not be sufficient to make the bats feel safer in their roosting and foraging environment. Egyptian fruit bats may require a group of more than two individuals to see a significant reduction in vigilance and an increase in foraging. While bats visited the food patch more frequently alone than when paired, this difference appeared to be due solely to the two pregnant females, an outlier pair, in the first set.

By multiple metrics, bats foraged significantly more when paired with a member of the opposite sex than with a member of the same sex. Similar differences in foraging across sex pairings have been observed in other species. For example, after observing the foraging of a same- or opposite-sex conspecific, zebra finches (*Taeniopygia guttata*) utilized social information differently based on the sex of the pairing [53]. Egyptian fruit bats may similarly change how they observe their companion and utilize learned patch information when paired with a member of the opposite sex, leading to more efficient foraging in this context. It could be that females are more neophobic than males, and that seeing the males feed from the novel source reduces their neophobia through social transmission of food-related information.

The set of four individuals involved in an experimental block significantly affected all foraging metrics. This result suggests that bat personality and potentially previous experiences among the individuals play a significant role in social foraging. The compatibility of foragers is a factor in their ability to cooperate in a foraging context. The individual members of a social group are important regarding the use of environmental and social information for making foraging decisions [54]. Egyptian fruit bats develop familiarity with some individuals over others and prefer familiar over unfamiliar conspecifics [13]. It follows that they would display different comfort levels with preferred individuals over nonpreferred individuals.

## 5. Conclusions

Egyptian fruit bats demonstrate optimal foraging and a high level of vigilance when foraging. In a social context, foraging behaviors were influenced by both the sex and identity of companions. Thus, the foraging efficiency of the Egyptian fruit bats in our experiment was largely mediated by the sex of individuals and the compatibility of the group. This species of bat is commonly used as an animal model in laboratory settings to study the neural basis of behavior; thus, it is paramount to understand the complex social interactions that may affect the relationships across individuals. These results will inform our future work regarding the choice of pairs during experiments on captive animals.

## Figures and Tables

**Table 1:** Linear models (ANCOVAs) demonstrate the effect of time spent foraging, number of patch visits, and mean patch visit time on giving-up densities in the Egyptian fruit bat. Values of *p* < 0.05 are bolded.

	Effect on GUDs				
	Df	Sum Sq	Mean Sq	F Value	Pr(>F)
Foraging time					
Sex	1	6.39	6.39	4.71	**0.04**
Foraging time	1	5.64	5.64	4.16	**0.04**
Sex: Foraging time	1	2.96	2.96	2.18	0.15
Residuals	32	43.40	1.36		
Number of patch visits					
Sex	1	3241.3	3241.3	6.05	**0.02**
Visit number	1	8075.2	8075.2	15.06	**<0.001**
Sex: Visit number	1	1871	1871	3.49	0.07
Residuals	32	17,155.3	536.1		
Mean visit time					
Sex	1	6.39	6.385	4.46	**0.04**
Mean visit time	1	4.98	4.98	3.47	0.07
Sex: Mean visit time	1	1.17	1.17	0.81	0.37
Residuals	32	45.86	1.43		

**Table 2:** Statistical output for generalized linear mixed models (GLMMs) and general linear models demonstrating the effect of social context (foraging in the absence or presence of a conspecific), pairing (male–male, male–female, or female–female), and the set of individual bats on time spent foraging, number of patch visits, and mean patch visit time. Where set or pairing was significant in the main model, we calculated relevant Tukey pairwise treatment comparisons. FF is female–female pairings, MM is male–male pairings, and MF is male–female pairings. Significant (*p* < 0.05) fixed effects and significant pairwise comparisons are bolded.

Response Variable	Model Type	Fixed Effects (Random Effects)	Statistics	Tukey Pairwise Comparisons	Statistics
Foraging time (s)	GLMM	Social	LRT_3_ = 0.43, *p* = 0.514	**Set1-Set2**	**z_64_ = 5.24**, ***p* < 0.001**
		Pairing	LRT_2_ = 1.18, *p* = 0.555	**Set1-Set3**	**z_64_ = 4.78**, ***p* < 0.001**
		**Set**	**LRT_1_ = 15.73**, ***p* < 0.001**	Set2-Set3	z_64_ = −0.56, *p* = 0.841
		(Bat ID)	Var. < 0.001, Std.Dev < 0.001		
Patch visit number	GLMM	**Social**	**LRT_3_ = 4.14**, ***p* = 0.042**	**Set1-Set2**	**z_64_ = 2.66**, ***p* = 0.021**
		**Pairing**	**LRT_2_ = 7.98**, ***p* = 0.019**	**Set1-Set3**	**z_64_ = 2.36**, ***p* = 0.048**
		**Set**	**LRT_1_ = 6.42**, ***p* = 0.040**	Set2-Set3	z_64_ = −0.33, *p* = 9.43
		(Bat ID)	Var. = 0.26, Std.Dev = 0.16	FF-MF	z_64_ = −1.10, *p* = 0.512
				FF-MM	z_64_ = 1.09, *p* = 0.512
				**MF-MM**	**z_64_ = 2.66**, ***p* = 0.022**
Mean visit time (s)	GLMM	Social	LRT_3_ = 1.08, *p* = 0.298	**Set1-Set2**	**z_64_ = 5.68**, ***p* < 0.001**
		**Pairing**	**LRT_2_ = 8.69**, ***p* = 0.013**	**Set1-Set3**	**z_64_ = 4.88**, ***p* < 0.001**
		**Set**	**LRT_1_ = 16.34**, ***p* < 0.001**	Set2-Set3	z_64_ = −0.98, *p* = 0.590
		(Bat ID)	Var. < 0.001, Std.Dev = 0.028	FF-MF	z_64_ = −1.10, *p* = 0.512
				FF-MM	z_64_ = 1.09, *p* = 0.519
				**MF-MM**	**z_64_ = 2.66**, ***p* = 0.022**
GUDs (g)	GLM	Social	LRT_3_ = 1.99, *p* = 0.159	**FF-MF**	**t_70_ = 2.65**, ***p* = 0.027**
		**Pairing**	**LRT_2_ = 7.77**, ***p* = 0.021**	FF-MM	t_70_ = 0.99, *p* = 0.587
		Set	LRT_1_ = 4.65, *p* = 0.098	MF-MM	t_70_ = −1.64, *p* = 0.237

## Data Availability

Data will be shared upon request.

## Supplementary Materials

The following supporting information can be downloaded at https://www.mdpi.com/article/10.3390/biology14121742/s1; Figure S1: Behavioral setup; Figure S2: Experimental food patches; Figure S3: Experiment 1 design; Video S1: Foraging bats.
