# Supplementary material for: Dinner Date: Opposite-Sex Pairs of Fruit Bats (Rousettus aegyptiacus) Forage More than Same-Sex Pairs
Source: Biology (Basel). 2025 Dec 5;14(12):1742. doi: 10.3390/biology14121742 (PMC12730429; doi:10.3390/biology14121742)
Supplement: Supplementary file 1 [file biology-14-01742-s001.zip › Supplementary material.docx]

**Supplementary material**


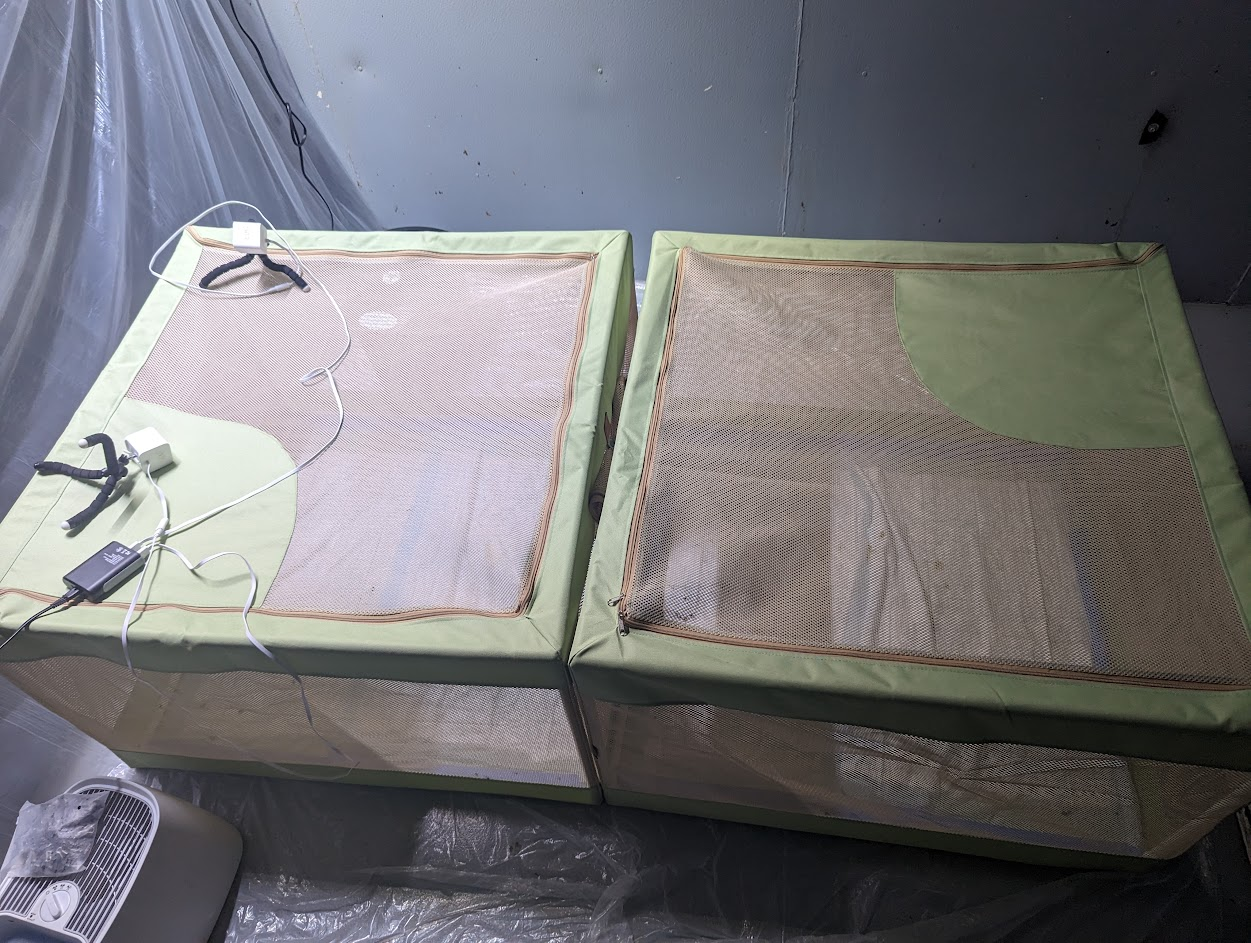


Supplementary Figure 1. Mesh enclosures with zippered openings used as partitions to allow for solitary or social foraging.


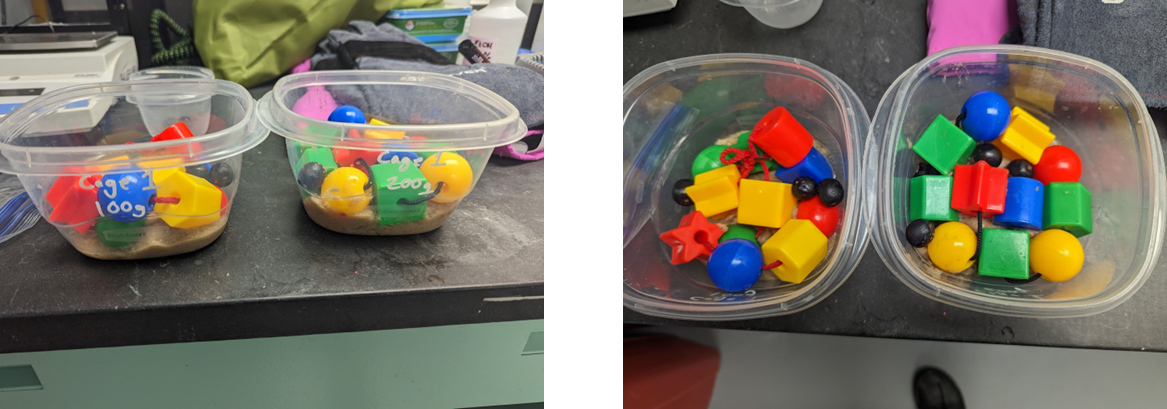


Supplementary Figure 2. Artificial food patches used to obtain giving-up densities in Egyptian fruit bats. These consisted of fruit smoothie as the food, 11 large beads tied together with shoestring as the substrate, and grapes on top to entice bats to forage. One patch contained 100 g of smoothie (“poor” patch) and the other 200 g of smoothie (“rich” patch).


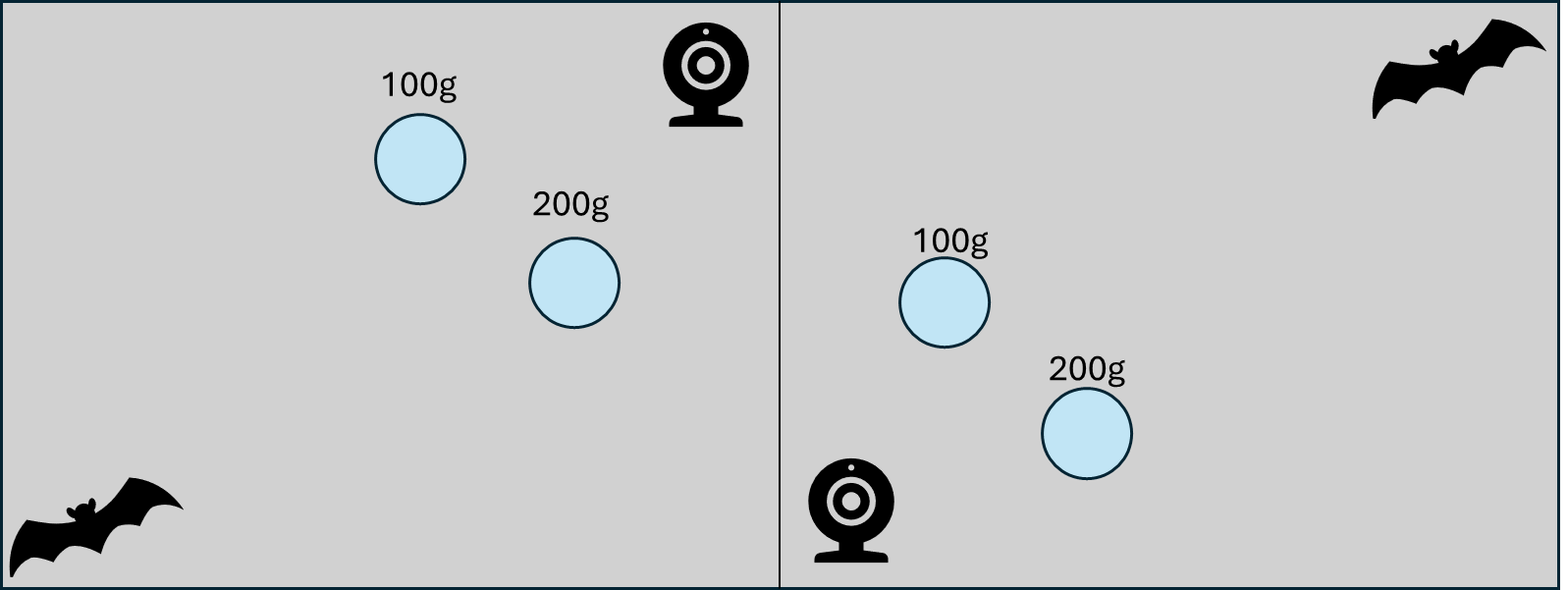


Supplementary Figure 3. Experiment 1 design for testing Egyptian fruit bat preference between 100g poor and 200g rich patches. Each half of the enclosure had one bat and one WAYZ camera pointed at two food patches (blue circles). For this experiment, bats were separated from each other via a mesh wall (middle divide) at all times.
